# Mapping Incidence and Prevalence Peak Data for SIR Modeling Applications

**DOI:** 10.1007/s00285-025-02299-6

**Published:** 2025-10-30

**Authors:** Alexander C. Murph, G. Casey Gibson, Lauren J. Beesley, Nishant Panda, Lauren A. Castro, Sara Y. Del Valle, Carrie A. Manore, Dave A. Osthus

**Affiliations:** 1 Statistics (CAI-4), Computing and Artificial Intelligence Division, Los Alamos National Lab, Los Alamos, 87545 New Mexico USA; 2 Information Systems & Modeling Group (A-1), Analytics, Intelligence, & Technology Division, Los Alamos National Laboratory, Los Alamos, 87545 New Mexico USA; 3 Theoretical Biology & Biophysics (T-6), Theoretical Division, Los Alamos National Laboratory, Los Alamos, 87545 New Mexico USA; 4 Information Sciences (CAI-3), Computing and Artificial Intelligence Division, Los Alamos National Laboratory, Los Alamos, 87545 New Mexico USA

**Keywords:** Compartmental Models, Disease Forecasting, Hospital Incidence, Prevalence, 62P10, 62P12

## Abstract

Infectious disease modeling and forecasting have played a key role in helping assess and respond to epidemics and pandemics. Recent work has leveraged data on disease peak infection and peak hospital incidence to fit compartmental models for the purpose of forecasting and describing the dynamics of a disease outbreak. Incorporating these data can greatly stabilize a compartmental model fit on early observations, where slight perturbations in the data may lead to model fits that forecast wildly unrealistic peak infection. We introduce a new method for incorporating historic data on the value and time of peak incidence of hospitalization into the fit for a Susceptible-Infectious-Recovered (SIR) model by formulating the relationship between an SIR model’s starting parameters and peak incidence as a system of two equations that can be solved computationally. We demonstrate how to calculate SIR parameter estimates – which describe disease dynamics such as transmission and recovery rates – using this method, and determine that there is a noticeable loss in accuracy whenever prevalence data is misspecified as incidence data. To exhibit the modeling potential, we update the Dirichlet-Beta State Space modeling framework to use hospital incidence data, as this framework was previously formulated to incorporate only data on total infections. This approach is assessed for practicality in terms of accuracy and speed of computation via simulation.

## Introduction

Compartmental models have seen broad usage at the onset of several disease outbreaks in the last century as a means to forecast expected numbers of infected individuals and to learn informative quantities about the disease such as transmission and recovery rates. Broadly speaking, a compartmental model describes the dynamics of a disease spread by breaking a population into set categories and modeling the process by which individuals transition through these categories. Perhaps the most basic is the Kermack–McKendrick model, often called the SIR or Susceptible-Infectious-Recovered model, which models the movement of subjects from being Susceptible, to Infected (and contagious), and then Removed from the system (Kermack et al. 1927). Since the development of the SIR model, further research has extended the idea to include additional compartments, such as the Exposed category in the SEIR model – where a subject has been exposed to the disease but is not yet infectious – and the ability to move back to the Susceptible category in the SEIS model (see Walter and Contreras (1999); Hethcote (2000) for an overview of each). Compartmental models have been applied to modeling tasks for the 2014-15 Ebola epidemic (Chretien et al. 2015), the 2009 A/H1N1 influenza pandemic (Nsoesie et al. 2014), several HIV outbreaks (Anderson 1988; Golub et al. 1993; Nyabadza et al. 2011), the recent COVID-19 pandemic (Zhao and Chen 2020; Cooper et al. 2020; Zhang et al. 2022), and many other epidemiological modeling tasks in the last century (see, for instance, Guanghong et al. (2004); LaDeau et al. (2011); Zhan et al. (2019) and references therein). In several applications of the SIR model, inference on the parameters is of central interest, as these parameters describe disease dynamics such as transmission and recovery rates (Burr and Chowell 2006; Melikechi et al. 2022).

The elegance of compartmental models is in their succinct ability to describe the state of a population in terms of how subjects transfer to and from the different categories, and thus fitting these models requires estimation of interpretable quantities such as rates of infection and recovery. For instance, denote the proportion of the population in the susceptible, infectious, and removed compartments by $S_t, I_t,$ and $R_t,$ respectively, such that $S_t + I_t + R_t = 1$ for all *t*. Then the SIR model is determined by the equations

1a $$\begin{aligned} \frac{dS_t}{dt}&= - \beta S_t I_t , \end{aligned}$$

1b $$\begin{aligned} \frac{dI_t}{dt}&= \beta S_t I_t - \gamma I_t , \end{aligned}$$

1c $$\begin{aligned} \frac{dR_t}{dt}&= \gamma I_t , \end{aligned}$$

where $\beta > 0$ is the disease transmission rate and $\gamma >0$ is the rate of recovery. If one knew these two rates, and the initial number of individuals in each category – $S_0, I_0$ and $R_0$ – the numbers $S_t, I_t, \text {and} \; R_t$ could be numerically simulated for any time-point *t*.

Some recent papers study SIR curve quantities of interest (QoIs) – such as the value of the peak of the infected curve and the limiting number of susceptible individuals – for inferential tasks related to modeling an epidemic (Miller 2009; Lang et al. 2018; Thompson et al. 2020). In Amaro (2023), the Gumbel distribution is suggested as a good approximation of the infection curve in an SIR model, and maps between the SIR curve parameters and historic data on infection count peak value/time are used to develop Method of Moments estimators of the Gumbel parameters. The Gumbel distribution is then used to approximate the exact solution of the SIR model. In Osthus et al. (2017), the relationship between peak infection value and the SIR parameters is used to incorporate historical data on epidemic peaks for the inferential task of fitting an SIR curve during the early stages of a seasonal epidemic, where previous years’ information on peak value and timing informs what might happen on a current year. The authors point out that an SIR curve is sensitive to small perturbations in the transmission and recovery rates, and that incorporating these data discourages models that well-represent early data but drastically over-predict the peak infection value. In McAndrew et al. (2024), both historic and surveyed QoIs are used to constrain an SEIRH model (where the added “H” category refers to people hospitalized at a given time) in much the same way as Osthus et al. (2017).

**Fig. 1:**
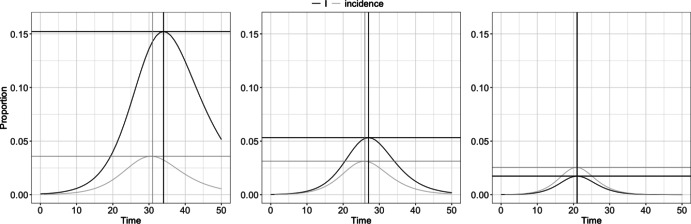
Three SIR models with incidence and starting values $S_0 = 0.9$ and $I_0 = R_0 = 0.05$. While it is possible for incidence to be greater than prevalence, this is not of practical interest

Incorporating historical peak values and times of an outbreak of a seasonal pathogen into a compartmental model is highly useful to accurately fit the model, especially early in the outbreak (see Osthus et al. (2017), Osthus et al. (2019), and McAndrew et al. (2024)), given that the pathogen is seasonal (so historical data on peak values and times may inform forecasts on a current outbreak) and is not an emerging pathogen (so therefore historical peak information is available). Using these historical QoIs to constrain a model fit requires determining a relationship between historical peak values/times and the parameters/initial conditions of the compartmental model (e.g., the SIR model). The challenge lies in this relationship. Current analytic solutions to the SIR model use peak information on the number of infected individuals at any given time, called *prevalence*, which is typically an unobserved quantity (Noordzij et al. 2010). Rather, most modern monitoring systems approximate the daily number of *new* cases of a disease, called *incidence*; analytic maps between peak value and time of incidence and the SIR curve parameters are currently unknown. For examples of the the differences between prevalence and incidence, see Fig. 1. Despite being principally unsound, the data application in Osthus et al. (2017) treated incidence data as if it were prevalence in order to use the known analytic relationships. There are several other papers (some modern papers) that fit $I_t$ to prevalence data rather than incidence data (Yorke and London 1973; Earn et al. 2000; Pollicott et al. 2012; Schmitt 2022,where the Pollicot paper fits using both types of data).

In this paper, we make three contributions. First, we develop the methods to map peak value and time of *incidence* to the parameters of the SIR model, given the initial conditions. Second, we use these maps to estimate the *basic reproduction number* of an SIR curve, and show how incorrectly using prevalence data in place of incidence data leads to a noticeable loss in accuracy. This is of particular note, as several authors have observed that misspecifying an epidemic model affects the accuracy of quantitative results (Wearing et al. 2005; Hart et al. 2019). Lastly, we demonstrate how to incorporate these new mappings into a forecasting model that can be fit on early observations of a seasonal disease outbreak, and provide the code used to do so. For the purposes of this paper, a “forecast” is taken to mean an unconditional statement about the future (in contrast to a “projection,” which is a conditional statement about the future). A key takeaway of this paper is that if SIR parameter inference is desired, then mistaking incidence data for prevalence data will result in biased estimation.

This paper is outlined as follows. In Section 2, we review existing methods for mapping SIR parameters to peak/time of prevalence. In Section 3, we develop identical maps for incidence, then provide methods for inverting these maps for both incidence and prevalence. In Section 4, we demonstrate how to perform inference on the basic reproduction number, and investigate the issues with treating prevalence data as incidence data. In Section 5, we provide a modeling framework to incorporate historic incidence peak/time data when fitting a disease forecasting model. We apply this model to influenza data in Section 6.

## Analytic Solutions to the SIR Equations for Incorporating Prevalence Data

The system (1a)-(1c) can be solved analytically for all time by reparameterizing the time axis to be instead in terms of the number of individuals removed from the system beyond the initial amount in the Removed category $R_0$,

2a $$\begin{aligned} S_t&= S_0 e^{-\beta \tau _t} , \end{aligned}$$

2b $$\begin{aligned} I_t&= S_0 + I_0 - S_0 e^{-\beta \tau _t} - \gamma \tau _t , \end{aligned}$$

2c $$\begin{aligned} R_t&= R_0 + \gamma \tau _t, \end{aligned}$$

where $\tau _t$ is the inverse of the map

3 $$\begin{aligned} t_{\tau } := \int _{0}^\tau \left[ \frac{d\tau '}{S_0 + I_0 - S_0 e^{\beta \tau '} - \gamma \tau '} \right] ^+, \end{aligned}$$

where $[x]^+:= \max \{x,0\}.$ The above form for the SIR dynamics is particularly useful since it allows one to calculate the number of individuals in each category without numerically simulating the entire system. The major drawback of this form is that mapping values back to an interpretable time requires one to approximate the integral in (3), since this integral is nonelementary. There exist other methods that derive an analytic solution to the SIR system (Harko et al. 2014). We use this formulation since it is well-known (having been originally developed in Kendall (1956)), and because it simplifies the calculation of SIR curve QoIs, such as peak prevalence value (PPV) and peak prevalence time (PPT). For instance, the maximum of (2b) with respect to $\tau$ occurs at

4 $$\begin{aligned} \tau ^* = \frac{1}{\beta } \log \left( \frac{\beta S_0}{\gamma } \right) , \end{aligned}$$

which can be derived via straightforward calculus. PPT can then be approximated from (3), and PPV calculated from (2b).

The process above of calculating PPV and PPT, denoted $(I_{t_{\tau ^*}}, t_{\tau ^*}),$ from the initial starting parameters can be reversed for fixed $S_0, I_0, R_0$. That is, given $(I_{t_{\tau ^*}}, t_{\tau ^*})$, we propose a method to calculate $(\beta , \gamma )$. Define $\rho$ as

5 $$\begin{aligned} \rho = \frac{\beta }{\gamma }. \end{aligned}$$

The quantity $\rho$ is more widely known as the *basic reproduction number*, which measures the average number of additional infections generated by a single new infection (Cadoni and Gaeta 2020). Plugging (4) into (2b) gives

6 $$\begin{aligned} I_t = S_0 + I_0 - \frac{1}{\rho } - \frac{\log (\rho S_0)}{\rho }. \end{aligned}$$

From here, setting $I_t:= I_{t_{\tau ^*}}$ and solving for $\rho$ is a (piecewise) convex optimization problem, which is typically fast and accurate computationally. Note that a change of variables for (3) gives

7 $$\begin{aligned} \beta t_{\tau } = \int _{0}^{\beta \tau } \left[ \frac{d\hat{\tau } }{S_0 + I_0 - S_0 e^{-\hat{\tau }} - \hat{\tau } / \rho }\right] ^+ , \end{aligned}$$

and that $\beta \tau ^* = \log (\rho S_0)$. Thus, once the value of $\rho$ is approximated, (7) can be evaluated to get $\beta t_{\tau ^*}$. Since $t_{\tau ^*}$ is assumed known *a priori*, an estimate for $\beta$ and $\gamma$ can be obtained via arithmetic. Uncertainty on these estimates can be obtained via simulation.

We introduce the above derivations not only because they will be used in the next Section, but also to motivate the type of calculation we aim to develop in this paper. While the above approach still requires computational methods to map from PPV and PPT to the SIR model parameters, this approach is much more direct than the brute force method of simulating several $(\beta , \gamma )$ combinations until a prevalence curve with a peak sufficiently near $(I_{t_{\tau ^*}}, t_{\tau ^*})$ is discovered (Prangle 2016).

## Mapping Incidence Data to SIR Parameters

As mentioned above, a primary focus of this work will be to develop maps between peak incidence value (PIV) and peak incidence time (PIT) and the initial SIR curve parameters. In this direction, consider the formulation of prevalence at a discrete time *t*,

8 $$\begin{aligned} I_t&= I_{t-1} + \beta S_{t-1} I_{t-1} - \gamma I_{t-1} . \end{aligned}$$

Putting (8) into words, prevalence is equal to the prevalence at the last time step, plus those in the infected category that infect those in the susceptible category, minus the number in the infected category that are removed from the system. The form for prevalence in (8) is particularly useful for this application since it contains a term that explicitly models incidence at time *t*: $\beta S_{t-1} I_{t-1}$.

### Mapping SIR Parameters to Peak Incidence Value and Time

Reparameterizing the time axis for the term $\beta S_{t} I_t$ (which we get from (8) as a term that gives the incidence at time $t+1$) using (2a) and (2b) gives the following form

9 $$\begin{aligned} \beta (S_0 e^{-\beta \tau _t}) (S_0 + I_0 - S_0 e^{-\beta \tau _t} - \gamma \tau _t ). \end{aligned}$$

The value for $\tau$ that maximizes (9) also satisfies the equation,

10 $$\begin{aligned} -(S_0 + I_0) + \gamma \tau + 2S_0 e^{-\beta \tau } - \frac{1}{\rho } = 0. \end{aligned}$$

Solving (10) is also a convex optimization problem on a single parameter, and is thus feasible and accurate to do numerically. A solution to (10) gives the $\tau$ for the timepoint directly *before* the time of max incidence, $\tau _{t^*-1}$, where $t^*$ denotes the timepoint of max incidence. Of course, this is all that is required to calculate the PIV, $\beta S_{t-1} I_{t-1},$ by a direct application of (9). The calculation of PIT is similarly straightforward. Using (2b), the prevalence at $\tau _{t^*-1}$ can be directly calculated. The prevalence value for $\tau _{t^*-1}$ and (2c) then gives $\tau _{t^*},$ and (3) can then be used to calculate $t^*$,

$$\begin{aligned} t^* = \int _{0}^{\tau _{t^*}} \left[ \frac{d\tau '}{S_0 + I_0 - S_0 e^{\beta \tau '} - \gamma \tau '} \right] ^+. \end{aligned}$$

### Mapping Peak Incidence Value and Peak Incidence Time to SIR Parameters

The proposed method to map PIV and PIT back to the parameters of an SIR curve solves the following system of equations implied by (9) and (10):

11 $$\begin{aligned} \beta (S_0 e^{-\beta \tau ^\star }) (S_0 + I_0 - S_0 e^{-\beta \tau ^\star } - \gamma \tau ^\star )&= \text {PIV} \end{aligned}$$

12 $$\begin{aligned} -(S_0 + I_0) + \gamma \tau ^\star + 2S_0 e^{-\beta \tau } - \frac{1}{\rho }&= 0 , \end{aligned}$$

where $\tau ^\star$ solves the equation

13 $$\begin{aligned} \int _{0}^{\tau ^\star } \left[ \frac{d\tau '}{S_0 + I_0 - S_0 e^{\beta \tau '} - \gamma \tau '} \right] ^+ = \text {PIT}. \end{aligned}$$

While solving a system of two equations with two unknowns (for $\beta$ and $\gamma$) is generally feasible computationally, the major bottleneck for this problem is the need to invert (13). A brute-force computational approach to solving this system of equations would require one to both invert and solve (13) for every value of $(\beta , \gamma )$ investigated. While experiments in this direction have proven to be surprisingly fast and accurate, the confounding computational approximations encourage a more analytic solution, or alternative computational strategies. We consider three possible alternatives to computationally estimating the integral (referred to as the “Compute Integral” method in the subsequent).

#### Taylor Approximation

Given a candidate $(\beta , \gamma )$ in any numerical solver, we approximate $\tau ^\star$ by taking the second degree Taylor expansion of the integral in (13) and solving for $\tau ^\star$ algebraically (Murray 2007). This leads to the following closed-form approximation,

14 $$\begin{aligned} \tau ^\star = \frac{\beta ^2}{S_0} \left[ \left( \rho S_0 - 1 \right) + \kappa \tanh \left( \frac{\gamma \kappa (\text {PIT})}{2} - \phi \right) \right] + R_0, \end{aligned}$$

where

$$\begin{aligned} \kappa&= \sqrt{\left( S_0 \rho - 1 \right) ^2 + 2 S_0 I_0 \rho ^2 }, \\ \phi&= \frac{1}{\kappa } {{\,\textrm{arctanh}\,}}\left[ S_0 \rho - 1 \right] . \end{aligned}$$

Using this approximation for $\tau ^\star$, we numerically solve the system of equations expressed by (11) and (12).

#### Single ODE Approximation

Instead of using a Taylor approximation to estimate $\tau ^\star$ it is possible to do so by numerically solving an Ordinary Differential Equation (ODE). Using the definition of $\tau$, we combine equations (2a) and (2c) to get

15 $$\begin{aligned} S_t = S_0 \exp \left( -\rho ( R_t - R_0 ) \right) . \end{aligned}$$

From here, we use (1c) and the assumption that $I_t = 1 - S_t - R_t$ to get the ODE,

16 $$\begin{aligned} \frac{dR_t}{dt} = \gamma \left[ 1 - S_0 e^{-\rho (R_t - R_0)} - R_t \right] . \end{aligned}$$

The authors in Cadoni and Gaeta (2020) point out that while (16) is a transcendental equation, it can be solved numerically, and has a single, unique solution by the general existence and uniqueness theorem for the solutions of ODEs. Using this approximation for $\tau ^\star$, we numerically solve the system of equations expressed by (11) and (12).

#### Full ODE Approximation

As a final computational method for mapping PIV and PIT to $\beta$ and $\gamma$, we numerically solve the system of ODEs described in (1a) - (1c). In an optimization algorithm, this would require the ODE to be solved for every possible $(\beta , \gamma )$ pair queried. Much like the brute force computational method, we expect this method to be accurate, but to come at a higher computational cost than the Single ODE and Taylor approximations.

#### Comparison via Simulation

We compare all the above approaches via simulation, repeating the following steps 1000 times:

1. Sample a (PPV, PPT) from the bivariate normal $\mathcal {N}(\mu , \Sigma )$ where $\mu = (0.0144, 17.9)$,
  $$ \Sigma = \begin{pmatrix} 0.000036 & 0.0187 \\ -0.0187 & 16.09 \end{pmatrix}, $$
  truncated so that PPV $\in (\theta _{I_0}, 1)$ and PPT $\in (1,35)$. This corresponds to the set of feasible values and sampling distribution described in Osthus et al. (2017);
2. Use the method from Section 2 to map this back to $(\beta , \gamma )$, then numerically simulate the system in (1a)-(1c) to get the “true” values for PIT and PIV;
3. With PIT and PIV, use each method described above to find approximate values $(\hat{\beta }, \hat{\gamma })$;
4. For all approximation methods, compare the estimated $\widehat{\text {PIT}}$ and $\widehat{\text {PIV}}$ (gotten by numerically simulating the system from the appropriate $(\hat{\beta }, \hat{\gamma })$) against the true PIT and PIV.

We outline the results from the above simulation in Table 1. While the fastest method is the Taylor approximation, this method is also the least accurate in estimating PIT. This is as expected, since this approximation is only accurate for sufficiently small values of $\rho (R_t - R_0)$ (Murray 2007). The Single ODE, Compute Integral, and Full ODE approximation methods are all mostly compareable, with Single ODE and Compute Integral being slightly more accurate in terms of PIV and the Full ODE achieving perfect estimation on PIT. Note that while the Single ODE and Full ODE methods are very similar, the Single ODE is occasionally off in predicting the PIT, while the full ODE always correctly predicts PIT. We suspect that this because the single ODE uses two numerical approximations: one to simulate an approximation for $\tau$ using (16), then another to solve the system of equations (11) and (12). This would also explain why the Full ODE method is faster than the Single ODE method.

While the Compute Integral method is the slowest of these approaches, all methods are very fast (each taking at most around half a second on average). Since the data sizes for PIT and PIV data are not exorbitantly large for the application in this paper, these computational burdens would be acceptable in converting a data set of $(\text {PIV}, \text {PIT})$ values to $(\hat{\beta }, \hat{\gamma })$ values using any of these methods. We recommend all methods except for the Taylor Approximation method for applications, since this method does so poorly with estimating PIT. Future work might examine alternative approximation methods, especially for approximating the inverse of the integral in (13). We use the Compute Integral method for the remainder of the analyses in this paper.

Since the results in Table 1 are all numerical approximations, it is possible that these results might change depending on which functional approximation method is used for each of these methods. For this paper, the optim function in R is used for all approximations (R Core Team 2024), using the method from Nelder and Mead (1965). There is one exception to this: when approximating proper time by inverting (13), the values for $\tau$ must be constrained between zero and one, so we used the approximation from Brent (1973), since this allows for constrained optimization. To demonstrate how these results might vary under alternative optimization schemes, we run this same code using the method from Broyden (1970) (with the exception that we keep the method from Brent (1973) when inverting (13)). These additional results are found in Appendix A.

**Table 1:** Errors and runtimes (in seconds) for the four approximation methods described in Section 3.2, using the simulation study described in Section 3.2.4. The methods are the Compute Integral approach (top of 3.2), the Taylor approximation (3.2.1), the single ODE approximation (3.2.2), and the full ODE approximation (3.2.3).

Quantity	Compute Integral	Taylor Approx.	Single ODE	Full ODE
Avg. PIV Error	1.810e$^{-4}$	1.472e$^{-4}$	1.838e$^{-4}$	1.197e$^{-3}$
Std. Dev. PIV Error	3.980e$^{-4}$	1.262e$^{-3}$	4.060e$^{-4}$	4.378e$^{-3}$
Avg. PIT Error	3.2e$^{-2}$	3.357	3.2e$^{-2}$	0
Std. Dev. PIT Error	1.761e$^{-1}$	1.899	1.761e$^{-1}$	0
Avg. Runtime	6.120e$^{-1}$	2.839e$^{-3}$	3.262e$^{-1}$	9.675e$^{-2}$
Std. Dev. Runtime	1.872e$^{-1}$	3.247e$^{-3}$	1.037e$^{-1}$	5.232e$^{-2}$

## Parameter Inference using Correctly Specified Data

We investigate inference on the SIR curve parameters $\rho$ using the map developed in Section 3.2 and data that are correctly specified as incidence. We further compare this against performing inference on these same parameters when incorrectly treating incidence data as prevalence. This investigation is performed via the following simulation study, which repeats the following 100 times:

1. Sample “true” parameters $(\beta , \gamma )$ (recording $\rho = \beta /\gamma$) and generate the corresponding SIR curve;
2. Generate data according to a Negative Binomial model, where the observation at the $t^\text {th}$ time point is distributed
  $$\begin{aligned} x_t \sim \operatorname {Negative Binomial}(i_t, r), \end{aligned}$$
  where $i_t$ – the true incidence value of the SIR curve – is taken as the mean, and *r* is the dispersion term (set to $r=100$ for this study);
3. Fit a smooth spline to the data (we used the gam function from the mcgv package in R (Wood 2003)). Record the value and time of the peak of the smoothed data;
4. Calculate $\hat{\rho }_{\text {inc}}$ using the map described in Section 3.2, which correctly specifies incidence data as incidence;
5. Calculate $\hat{\rho }_{\text {prev}}$ using the map described in Section 2, incorrectly specifying incidence data as prevalence;
6. Record the Mean Absolute Error (MAE) for these two parameter estimates.

**Fig. 2:**
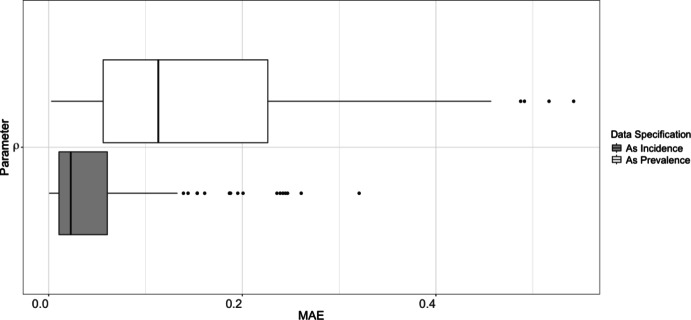
MAE of estimates on $\rho$ using (correctly) specified incidence data as incidence and using (incorrectly) specified prevalence data as incidence

The MAEs for the two parameter estimations are visualized via boxplots in Figure 2. These boxplots show that the estimation for $\rho$ is more accurate on average when incidence data are correctly specified as incidence, while using prevalence data in place of incidence leads to greater observed errors.

While misspecifying incidence data as prevalence data may lead to biased estimation, it is worth noting that poor estimation of $\rho$ may also be due to other issues, such as the choice for model structure. For instance, if there were data for which an SEIR would be more appropriate, using an SIR model instead may lead to poor estimation (even if the data are correctly specified). The example in this section only demonstrates that biased estimation occurs when misspecifying the data when these data are appropriately modeled by an SIR model.

## A Bayesian State-Space SIR Model

**Fig. 3:**
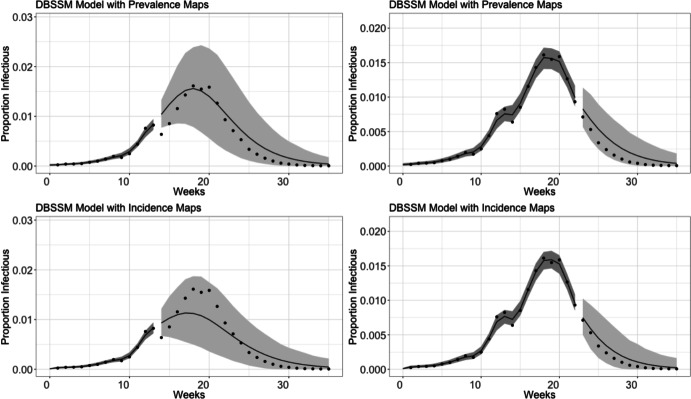
The DBSSM fit to the 2010 US nationwide influenza outbreak starting at week 13 (left column) and week 22 (right column). The dark grey regions correspond to the 95% credible regions of the posterior density while the light grey regions correspond to the 95% prediction intervals. The top plots reproduces the forecasting model of Osthus et al. (2017), which incorrectly treated incidence data as though it were prevalence data. The bottom plots fit the forecasting model described in Section 3, which correctly treats incidence data as incidence data. Earlier on in the outbreak, the model developed in this paper has a tighter prediction interval. The forecasts starting at week 22 are quite similar

In this section, we introduce the Dirichlet-Beta state-space model (DBSSM) from Osthus et al. (2017) and update it to incorporate historic PIV and PIT data. The original formulation of the DBSSM was to answer an observed issue associated with using the SIR model for early-pandemic forecasting tasks. Namely, that two SIR curves that reasonably fit early count data may lead to drastically different PPV predictions. In a simulation example, Osthus et al. (2017) show two such SIR curves that have peaks that differ by 30% of the entire population, even though they have a nearly-identical fit to the early-pandemic data observations (see Figure 3 in the cited paper). To address this stability issue, the DBSSM incorporates historic PPV and PPT data into the prior specifications to discourage SIR curve fits with peak values that are greatly above reasonable expectations. As mentioned previously, this incorporation of prevalence data is not the most practical approach, since incidence data is generally the observed quantity. A further shortcoming in the original DBSSM formulation is that it learns a map between PPT and the SIR curve parameters, rather than using an analytic map. After introducing this model, we will identify ways that the methodology developed in this paper will improve these issues for the DBSSM.

Let $y_t$ be the observed proportion in a population that tested positive for some disease at some timepoint *t*, and let $\theta _t = (S_t, I_t, R_t)'$. Then the DBSSM is defined as,

17 $$\begin{aligned} y_t | \theta _t, \phi \sim \text {Beta}\left( \lambda \text {In}_t, \lambda (1 - \text {In}_t) \right) \end{aligned}$$

18 $$\begin{aligned} \theta _t | \theta _{t-1}, \phi \sim \text {Dirichlet}\left( \iota f(\theta _{t-1}, \beta , \gamma ) \right) , \end{aligned}$$

where $\phi = \{S_0, I_0, R_0, \beta , \gamma , \lambda , \iota \}$, $\lambda , \iota$ are variance control parameters, and *f* is a map that propagates the SIR system determined by $(\theta _{t-1}, \beta , \gamma )$ forward one step according to (1a)-(1c). Note that, by this set up, the set of parameters $\theta _{0:t'} = \{ \theta _0, \theta _1, \dots , \theta _{t'} \}$ is a first-order Markov chain, and that for all $s\ne t$, the data observations $y_s$ and $y_t$ are independent given $\theta _t$. The variable $\text {In}_t$ denotes the *incidence* of the system at time *t*; in the original formulation of this model, the prevalence – $I_t$ – was used here. The incidence at time *t* is directly calculated using $\theta _t$ and (8).

The conditional expectations of the model described by (17) and (18) are unbiased,

19 $$\begin{aligned} E(y_t | \theta _t, \phi ) = \text {In}_t \end{aligned}$$

20 $$\begin{aligned} E(\theta _t | \theta _{t-1}, \phi ) = f(\theta _{t-1}, \beta , \gamma ) \end{aligned}$$

while their respective variances reduce to zero as $\lambda , \iota \rightarrow \infty$. Of course, the conditional mean in (20) is dependent upon the accuracy of *f* in propagating the latent space $\theta _{t-1}$ forward one time step. The authors in Osthus et al. (2017) used a fourth-order Runge-Kutta approximation and observed reasonable accuracy.

We review the full Bayesian framework of the DBSSM and provide the prior specification in Appendix B. The main innovation of this model is that the parameter space is expanded to include the latent variable $z = (PPT, PPV)$, and this latent variable is given a prior that incorporates historic PPT and PPV data. In the following, we review this prior, $\pi (z|\theta _0)$, and the mechanism by which this prior informs the SIR parameters $\beta$ and $\gamma$. These priors are then each updated according to the theory developed in this paper.

### Specification of $\pi (z | \theta _0)$

The prior on $\pi (z | \theta _0)$ in the DBSSM is a minimal-assumption distribution on historic data on PPT and PPV. Note that this is the mechanism by which the authors in Osthus et al. (2017) directly address the aforementioned stability issues with fitting an SIR curve with early pandemic data. They do so by fitting a normal distribution to historic influenza QoI data, truncated to enforce that an epidemic will occur (the lower bound on *PPV* was set to $I_0$) and so that the peak happens within the influenza forecasting season (*PPT* was required to be between the $1^{st}$ and $35^{th}$ weeks). While somewhat loose, this prior gives very small (or zero) probability to values of (*PPV*, *PPT*) that are drastically outside of historically observed pandemics.

In the formulation of the DBSSM developed in this paper, the same prior used on PPT and PPV is now used on PIT and PIV. To connect this constraint into the model, we must next define how the assumption on this latent space affects the learning of the SIR parameters $\beta$ and $\gamma$.

### Specification of $\pi (\beta , \gamma | z, \theta _0)$

With the addition of the latent variable *z*, the prior needed for the SIR parameters is $\pi (\beta , \gamma | z, \theta _0)$. In the original formulation of the DBSSM, this prior is reparameterized according to $(\rho , \gamma )$, then factorized. Thus, priors are instead given to $\pi (\rho | z, \theta _0)$ and $\pi (\gamma | \rho , z, \theta _0)$. This additional formulation is done to utilize the following analytic relationship from Weiss (2013),

21 $$\begin{aligned} PPV = g_1(S_0, I_0, \rho ) = I_0 + S_0 - S_0 \rho \left[ \log (S_0) + 1 - \log (S_0 \rho ) \right] . \end{aligned}$$

By inverting this relationship, samples from the latent quantity *z* immediately determine the corresponding value of $\rho$. The appropriate prior on this quantity would then be

22 $$\begin{aligned} \pi (\rho | z, \theta _0) ~\propto ~ \delta (S_0 \rho - g_1^{-1}(PPV, S_0, I_0)), \end{aligned}$$

where $\delta$ is the Dirac delta function. For the prior on $\gamma$, the map between $\gamma$ and PPT is estimated using a simulated data set of 5250 SIR curves. This map,

23 $$\begin{aligned} PPT = g_2(S_0, I_0, \rho , \gamma ), \end{aligned}$$

was then used in lieu of any analytic form, and the prior on $\gamma$ was set to

24 $$\begin{aligned} \pi (\rho | z, \theta _0) ~\propto ~ \delta (S_0 \rho - g_2^{-1}(PPV, S_0, I_0, \rho )), \end{aligned}$$

We reiterate that there are two major shortcomings to the above prior specifications. First, the above priors assume that there is access to historical PPV and PPT data, which is typically not the case, as public health data are generally on incidence, not prevalence. In the original paper, incidence data were used instead of prevalence data without explicit justification. Second, a map between PPT and the SIR parameters is estimated even though an analytic map between these quantities exists (see (3) and (4)), unnecessarily introducing a source of uncertainty.

The methods developed in this paper correct the limitations found in the original formulation of the DBSSM, since they provide maps to replace $g_1, g_2$ above with

25 $$\begin{aligned} (PIV, PIT) = h(S_0, I_0, \beta , \gamma ), \end{aligned}$$

where $h^{-1}$ denotes the algorithm discussed in Section 3.2. Thus, the joint prior used for $\beta , \gamma$ in this updated version of the DBSSM is

26 $$\begin{aligned} \pi (\beta , \gamma | z, \theta _0) ~\propto ~ \delta \left( || (\beta , \gamma ) - h^{-1}(PPV, S_0, I_0)||_1 \right) , \end{aligned}$$

where $||\cdot ||_1$ is the 1-norm.

The naive treatment of incidence data as prevalence data (as was done in Osthus et al. (2017)), need not necessarily lead to a loss of forecasting accuracy in the final model. An incidence curve can well approximate, or even be equivalent to, a prevalence curve. For instance, consider the SIR model constrained so that $\gamma = 1$, which corresponds to the Reed-Frost model (Abbey 1952). In this case, incidence is precisely equal to prevalence, and thus either method for incorporating historic QoI data should yield an equivalent model. This insight leads to the following Remark:

#### Remark 1

Using incidence QoI data in place of prevalence QoI data naively leads to an SIR curve where the *I* compartment – which normally corresponds to prevalence – now models the progression of incidence. Using historic incidence QoI data in the way outlined in this manuscript uses the incidence curve to model incidence as is desired. While misspecifying the data may not affect **forecasting**, this misspecification leads to **rate parameter estimates** that cannot be interpreted as infection and recovery rates. Of course, the model should be correctly specified (using incidence data as incidence data) whenever possible.

## Application to Seasonal Influenza Data

We recreate the data application from Osthus et al. (2017), using the updated model from Section 5. The aim of this application is not to improve the forecasting in the original formulation of the DBSSM (see Remark 1). Rather, we will demonstrate that the forecasting capabilities of this model remain the same, while we also observe different estimates for the infection rate $\beta$, the recovery rate $\gamma$ and the basic reproduction number $\rho$.

The source data modified and then used for this application are counts of patients seen in the US with an influenza-like illness (ILI), where ILI is defined as having a temperature of at least 100 degrees Fahrenheit, a cough and/or a sore throat, and no known cause for those symptoms other than influenza (CDC 2024). These data are collected weekly, where more than 3400 outpatient healthcare providers report to the CDC the number of patients with ILI they treated (CDC 2023).

The number of patients reported as having ILI will naturally also include cases of respiratory illnesses other than influenza. Following the approach of Shaman et al. (2013), we use virologic surveillance data (where patients are actually tested for influenza) to estimate the proportion of ILI patients with influenza, then multiply ILI data by this proportion. This corrected data is referred to as ILI+. For more details on this adjustment, see Shaman et al. (2013). Note that the ILI+ data estimates the weekly incidence of influenza cases – not prevalence.

To fit both versions of the DBSSM, we use ten influenza seasons: the seasons that started in the years 2002-2008, and the seasons that started during 2010-2013. The year 2009 was omitted since this year corresponds to a pandemic. We only use the data prior to flu season for which we are forecasting to train the prior for the DBSSM. Each season is defined as 35 consecutive Morbidity and Mortality Weekly Reports (MMWR) weeks starting on roughly the first week of October. MMWR weeks are defined as “the week of the epidemiologic year for which the National Notifiable Diseases Surveillance System disease report is assigned by the reporting local or state health department for the purposes of disease incidence reporting and publishing (Centers for Disease Control and Prevention 2025).” In this paper, MMWR week 40 is treated as $t = 1$).

For a estimated proportion of individuals in a population infected with influenza at timepoints $\mathbb {T} = \{ 1, \dots , T\}$, suppose only the ILI+ data up through $t' \in \mathbb {T}$ are observed. Given this, we simulate 62500 samples from the posterior $\pi (\varvec{\theta _{{1:t'}}}, \varvec{\phi } | y_{1:t'} )$ for four separate chains, discarding the first 12500 as burn-in and thinning out all but every tenth observation in the remaining samples. Given these draws from the posterior distribution, the posterior predictive density, $\pi (y_{(t'+1):T} | y_{1:t'})$, is used to estimate “future” observations of ILI+ data.

We perform two separate fits of this posterior model, on the first thirteen weeks ($t'=13$) and on the first twenty two weeks ($t' = 22$), for the considered ILI+ data for the 2010 influenza season in the United States. These fits are performed both using the original formulation of the DBSSM, which naively uses incidence data directly in place of prevalence data, and using the new formulation developed in this paper, which uses the new maps developed in Section 3 for a more principled treatment of incidence data. These fits and forecasts are outlined in Figure 3. The dark shaded grey regions prior to $t'$ mark the 95 percentiles of the posterior density, while the lighter grey shaded regions after $t'$ make the 95% prediction intervals. The inflection points in the dark grey regions are possible because the DBSSM model prior to the forecast model only uses the SIR model to determine the conditional mean (20); the predictions (the light grey regions) largely follow a fixed parameter value SIR model. The forecast using incidence data up until $t'=13$ has a narrower prediction interval than the one using prevalence data, but the mean forecast does not capture the truth as well; each of the forecasts that use data up through $t'=22$ are largely comparable. We perform a further investigation with an additional forecasting comparison (one on the 2014 influenza season) in Appendix C. We also perform a comparison of the model developed in this paper to another influenza forecasting model.

**Fig. 4:**
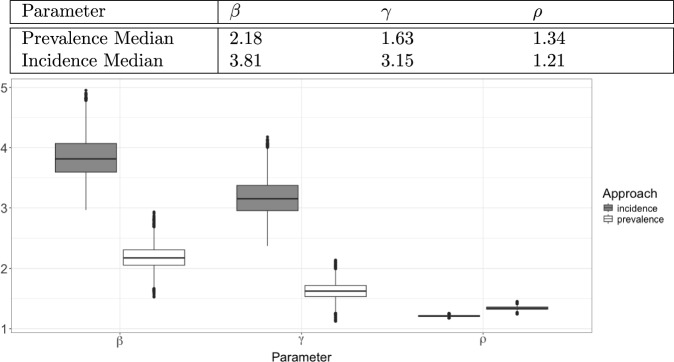
Infection rates, recovery rates, and reproductions numbers drawn from the Gibbs sampler used to fit the DBSSM using both specifications of incidence data. Using incidence data specified as prevalence leads to different estimates for these parameters. These values were calculated using the data up through timepoint 22 ($t'=22$)

This method also has strong implications for the interpretability of $\beta , \gamma$ for the fitted model. Indeed, only the updated version of the DBSSM developed in this paper leads to realizations of these parameters that can accurately be interpreted as the infection rate ($\beta$), recovery rate ($\gamma$), and the basic reproduction number ($\rho$). We observe how different the estimations are for each of these parameters under the misspecified data and under the correctly specified data in Figure 4. The posterior draws for the different data specifications are starkly different for each of the three parameters. Note that the absolute difference for the basic reproduction numbers is less than those observed for $\beta$ and $\gamma$; since small values of $\beta$ correspond to small values of $\gamma$ (and vice versa), the differences in the ratio are smaller. However, the basic reproduction numbers for the different data specifications are still practically very different. For instance, the proportion of the population that would need to be inoculated for influenza to avoid an outbreak would be $\sim 25\%$ according to the model that uses prevalence data as incidence data, while the model with correctly specified data predicts this number to be $\sim 17\%$.

## Discussion

The main contribution of this paper is the development of methods to map the time and value of peak incidence to the SIR curve parameters, and vice versa, for the purpose of forecasting tasks and inference on disease rate parameters. We do this by computationally solving a system of equations ((9) and (10)). There are several impactful uses of these maps in the context of previous literature. First, much like how the peak prevalence value (PPV) and time (PPT) are useful for public health response to an epidemic (Weiss 2013), the analogous quantities for incidence are also useful, since they describe the influx of new patients entering the hospital system on a given day (Lipsitch et al. 2011). Second, this work improves upon existing work that uses historical prevalence data to model epidemics by creating a map from PIT and PIV to the SIR parameters, since incidence is typically the data that is available for ongoing epidemics (Osthus et al. 2017; Amaro 2023). In the case of the application in Osthus et al. (2017), where incidence data were used in place of prevalence data without justification, we have shown that this leads to biased SIR parameter estimates (see Figure 4). Furthermore, our results indicate that forecasts performed using the erroneous data specification lead to larger prediction intervals earlier on in the outbreak, although this forecast is largely comparable for the correct specification later on in the outbreak (see Figure 3 and Remark 1). Of course, it remains more correct in principle to use incidence data appropriately when fitting compartment models for forecasting with ongoing incidence data (Nsoesie et al. 2013; Chowell et al. 2016; Abolmaali and Shirzaei 2021). We have provided a modeling framework that incorporates these data appropriately (see Section 5). Lastly, we have shown that when inference on the disease transmission and recovery rates is of interest, it is important to use the correctly specified data (incidence data as incidence data), since failing to do so will lead to a noticeable loss in accuracy for the parameter estimates.

We highlight that the forecasting methods introduced in this paper are most relevant for the outbreak of seasonal pathogens, since it is for these pathogens that past information on PIV and PIT would help inform what to expect during an ongoing outbreak. For other known pathogens, the behavior of the outbreak may vary too significantly to use past peak information. For emerging pathogens, there is no relevant information with which to use this methodology.

As a direction for future work, it would be useful to investigate better approximations for the solution to (13). The Taylor Approximation in Section 3.2.1 was by far the fastest computationally, but it came with the highest error on PIT. Finding a fast and accurate approximation to this equation would greatly increase the runtime for applications where the map between the SIR parameters and PIT/PIV must be evaluated several hundreds of thousands of times. However, for most applications (including the one in this paper), the Compute Integral approximation is sufficiently fast.

A further area of future work is extending these methods to more sophisticated compartmental models (e.g. the SEIR or SEIRH model). In addition to being a difficult task analytically, approaches to doing this would likely have to incorporate some additional form of information, as (PIV, PIT) are only two values that are then meant to determine a model with three or more parameters. Either another QoI would need to be incorporated, or several solutions would need to be proposed via simulation. With these added capabilities, it would also be interesting to try the improved model described in this paper on several other data applications on a wide range of seasonal pathogens.

## Figures and Tables

**Fig. 5:**
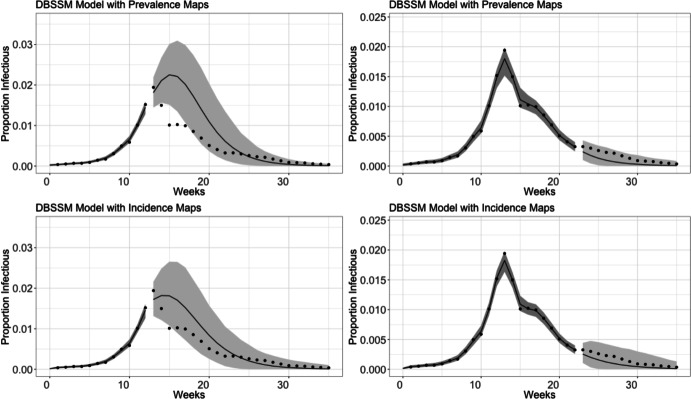
The DBSSM fit to the 2014 US nationwide influenza outbreak starting at week 13 (left column) and week 22 (right column). The dark grey regions correspond to the 95% credible regions of the posterior density while the light grey regions correspond to the 95% prediction intervals. The top plots reproduces the forecasting model of Osthus et al. (2017), which incorrectly treated incidence data as though it were prevalence data. The bottom plots fit the forecasting model described in Section 3, which correctly treats incidence data as incidence data

**Fig. 6:**
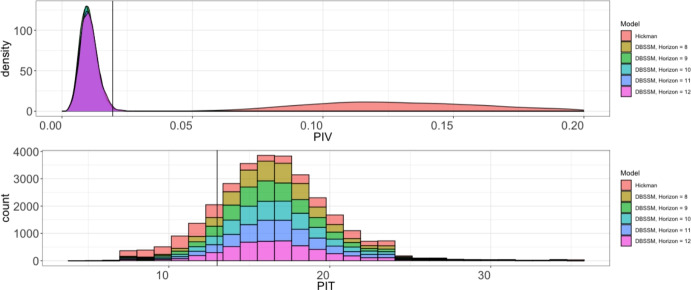
The DBSSM fit to the 2014 US nationwide influenza outbreak starting at weeks 8-12 compared against the model from Hickmann et al. (2015). The top plot visualizes predictions of PIV from each model; the bottom plot visualizes predictions of PIT

**Fig. 7:**
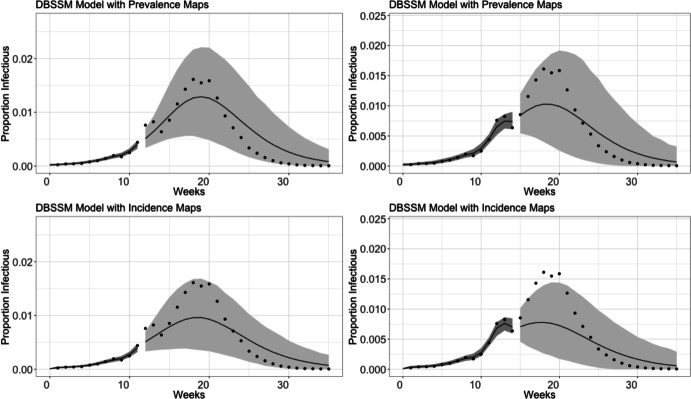
The DBSSM fit to the 2010 US nationwide influenza outbreak starting at week 11 (left column) and week 14 (right column). The dark grey regions correspond to the 95% credible regions of the posterior density while the light grey regions correspond to the 95% prediction intervals. The top plots reproduces the forecasting model of Osthus et al. (2017), which incorrectly treated incidence data as though it were prevalence data. The bottom plots fit the forecasting model described in Section 3, which correctly treats incidence data as incidence data

## Appendix A Solving for SIR with an Alternative Numerical Approximation Method

The results from the different numerical approximations in Table 1 are dependent on the algorithm used for integration and the minimizations. To demonstrate how these results might vary under alternative optimization schemes, we run this same code using the method from Broyden (1970) (with the exception that we keep the method from Brent (1973) when inverting (13)). These results are outlined in Table 2. The conclusions in Section 3.2.4 remain unchanged.

**Table 2:** Errors and runtimes (in seconds) for the four approximation methods described in Section 3.2, using the simulation study described in Section 3.2.4, using the alternative numerical method described in Broyden (1970). The methods are the Compute Integral approach (top of 3.2), the Taylor approximation (3.2.1), the single ODE approximation (3.2.2), and the full ODE approximation (3.2.3).

Quantity	Compute Integral	Taylor Approx.	Single ODE	Full ODE
Avg. PIV Error	8.987e$^{-3}$	2.718e$^{-3}$	8.914e$^{-3}$	1.355e$^{-3}$
Std. Dev. PIV Error	1.235e$^{-2}$	5.858e$^{-3}$	1.233e$^{-2}$	4.601e$^{-3}$
Avg. PIT Error	1.060e$^{-1}$	3.395	1.04e$^{-1}$	0
Std. Dev. PIT Error	3.112e$^{-1}$	2.064	3.054e$^{-1}$	0
Avg. Runtime	1.976	6.983e$^{-3}$	1.053	9.629e$^{-2}$
Std. Dev. Runtime	6.351e$^{-1}$	4.004e$^{-3}$	3.456e$^{-1}$	5.224e$^{-2}$

## Appendix B Specification of the Dirichlet-Beta State-Space Model

The DBSSM is fit using a fully Bayesian framework, where the interest lies in estimating the joint posterior density of the latent space $\theta _{1:t'}$ and $\phi$ given the observed data $y_{1:t'}$. Using the conditional independence assumption described above, this density can be written as follows:

B1 $$\begin{aligned} \pi (\theta _{1:t'}, \phi | y_{1:t'} )~ \propto ~ \pi (\phi ) \pi (y_{1:t'}, \theta _{1:t'} | \phi ) = \pi (\phi ) \prod _{t = 1}^{t'} \mathcal {L} (y_t | \theta _t, \phi ) \pi (\theta _t | \theta _{t-1}, \phi ), \end{aligned}$$

where $\pi (\phi )$ is some prior on $\phi$, $\mathcal {L} (y_t | \theta _t, \phi )$ is the data likelihood determined by (17), and the distribution $\pi (\theta _t | \theta _{t-1}, \phi )$ is determined by (18). To perform forecasts on observations $y_{t': T}$, where *T* is the final timepoint of the outbreak, one uses the posterior predictive distribution, where the model and latent-space parameters are integrated out:

B2 $$\begin{aligned} \pi (y_{ (t'+1):T} | y_{1 : t'} ) = \int \int \pi (y_{ (t'+1):T}, \theta _{1:T}, \phi | y_{1 : t'} ) d\theta _{1:T}, d\phi . \end{aligned}$$

To complete the specification of the DBSSM, one must determine what priors to put on the model parameters in $\phi$. It is here that the authors in Osthus et al. (2017) directly address the aforementioned stability issues with fitting an SIR curve with early pandemic data. Define the latent variable $z = (PPT, PPV)$, and let $\varvec{\phi }$ be the expanded model parameter vector that includes *z*. We factorize the new prior distribution on $\varvec{\phi }$ to get

B3 $$\begin{aligned} \pi (\varvec{\phi }) = \pi (\iota ) \pi (\lambda | \iota ) \pi (\theta _0 | \lambda , \iota ) \pi (z | \theta _0, \lambda , \iota ) \pi (\beta , \gamma | z, \theta _0, \lambda , \iota ). \end{aligned}$$

Several modeling assumptions on this conditional distribution give the abbreviated form,

B4 $$\begin{aligned} \pi (\varvec{\phi }) = \pi (\iota ) \pi (\lambda ) \pi (\theta _0) \pi (z | \theta _0) \pi (\beta , \gamma | z, \theta _0). \end{aligned}$$

Specification of the individual distributions in (B4) is what remains to fully define the DBSSM. The priors $\pi (\iota ), \pi (\lambda ),$ and $\pi (\theta _0)$ remain unchanged and can be found in the original paper. In Section 5, the priors on $\pi (z | \theta _0)$ and $\pi (\beta , \gamma | z, \theta _0)$ are described and updated, when necessary, with the theory developed in this paper.

## Appendix C Second Application to Influenza Forecasting and Model Comparison

We apply the DBSSM model from Section 6 to forecast the 2014 influenza season in the United States. The fits are performed both using the original formulation of the DBSSM and the new formulation that correctly specifies incidence data. These fits and forecasts are outlined in Figure 5. In this figure, the model built using correctly-specified incidence data correctly turns downwards sooner than the model built with incorrectly-specified data.

We next consider two additional forecasting weeks on the 2010 US nationwide influenza outbreak starting at weeks 11 and 14 in Figure 7. The results are largely comparable to those of Figure 3, though the model with correctly specified incidence struggled with the downturn observed at week 14. We maintain, however, that the parameter inference for this model is the only interpretable case.

The main aim of this paper was not to argue for better (such as in Fig 5) or worse (such as in Fig 7) forecasting between a correctly-specified and an incorrectly-specified model (see Remark 1). We were primarily interested in the appropriate estimation of the parameters in the compartmental model. The forecasting performance was more appropriately assessed in the original paper on the DBSSM model.

We compare the model developed in this paper’s ability to predict PIT and PIV for the 2014 influenza season against the prior model described in Hickmann et al. (2015). In the model from Hickmann et al. (2015), a mean and standard deviation of ILI+ for every MMWR week is calculated from a dataset that contains every flu season prior to the current season. From these predictions, individual ILI+ trajectories can be simulated from independent normal distributions at each MMWR week. Via Monte Carlo, a distribution of PIT and PIV predictions can be simulated, and compared against the PIT and PIV predictions from this paper. We compare the model from Hickmann et al. (2015) against the DBSSM using incidence data as incidence at several forecasting horizons in Figure 6. Note that the Hickman model does not use any data from the current influenza season, while the model in this paper does. For this reason, we include instances of the DBSSM fit to very early horizons (starting at MMWR week 8). The DBSSM is much better at estimating PIV than the Hickman model, while both models are largely comparable in predicting PIT (with the Hickman model giving slightly more weight near the truth).

## Declarations

### Code Availability

All software used to perform the simulations and studies in this paper are publicly available at https://github.com/lanl/precog.
